# Evaluation of a New Automated Mono-Test for the Detection of *Aspergillus* Galactomannan: Comparison of *Aspergillus* Galactomannan Ag VirCLIA^®^ Mono-Test with Platelia^TM^ *Aspergillus* Ag ELISA Assay

**DOI:** 10.3390/jof10110793

**Published:** 2024-11-15

**Authors:** Giuliana Lo Cascio, Valentina Lepera, Annarita Sorrentino, Domenico Caleca, Paolo Gigante, Gabriella Tocci, Alda Bazaj, Annalisa Mancini, Marina Bolzoni, Evelina Cattadori, Davide Gibellini, Chiara Gorrini, Claudio Farina, Roberta Schiavo, on behalf of the Medical Mycology Committee (CoSM)—Italian Association of Clinical Microbiology (AMCLI)

**Affiliations:** 1 Clinical Microbiology and Virology Unit, Azienda USL di Piacenza, 29121 Piacenza, Italy; v.lepera@ausl.pc.it (V.L.); g.tocci@ausl.pc.it (G.T.); c.gorrini@ausl.pc.it (C.G.); r.schiavo@ausl.pc.it (R.S.); 2 Department of Medicine and Surgery, University of Parma, 43125 Parma, Italy; 3 Medical Mycology Committee (CoSM)—Italian Association of Clinical Microbiology (AMCLI), 20159 Milano, Italy; 4 Clinical Microbiology and Virology Unit, Azienda Ospedaliera Universitaria Integrata, 37134 Verona, Italy; annarita.sorrentino@aouvr.veneto.it (A.S.);; 5 Quality & Research Unit, Azienda USL di Piacenza, 29121 Piacenza, Italy; a.mancini@ausl.pc.it (A.M.); m.bolzoni@ausl.pc.it (M.B.); e.cattadori@ausl.pc.it (E.C.); 6 Clinical Microbiology and Virology Unit, ASST Papa Giovanni XXIII, 24127 Bergamo, Italy

**Keywords:** Galactomannan Ag, Platelia Aspergillus, VirClia *Aspergillus* Galactomannan antigen

## Abstract

The analytical performance of the new *Aspergillus* Galactomannan Ag VirCLIA^®^ mono-test (Vircell S.L.) was compared to the Platelia™ *Aspergillus* Ag ELISA assay (Bio-Rad). Prospective serum and bronchoalveolar lavage (BAL) samples from patients at risk of invasive aspergillosis (IA) were tested using both the *Aspergillus* Galactomannan Ag VirCLIA^®^ mono-test and the Platelia™ *Aspergillus* Ag ELISA assay. Concordance, sensitivity, specificity, and positive and negative predictive values were calculated using the manufacturer-recommended cutoff levels. Receiver operating characteristic (ROC) analysis and the Youden index were performed to determine the optimal cutoff. A total of 187 serum samples and 73 BAL samples were analyzed with both assays. The concordance between the *Aspergillus* Galactomannan Ag VirCLIA^®^ mono-test and the Platelia™ *Aspergillus* Ag ELISA assay was 87.8%, with a Cohen’s kappa of 0.75. The sensitivity and specificity of the *Aspergillus* Galactomannan Ag VirCLIA^®^ mono-test were 78.6% and 96.2%, respectively, with positive and negative predictive values of 94.8% and 83.3%. The ROC curve for the *Aspergillus* Galactomannan Ag VirCLIA^®^ mono-test demonstrated an area under the curve (AUC) of 0.87, and the Youden index at the manufacturer’s established cutoff was 0.73. This new *Aspergillus* Galactomannan Ag VirCLIA^®^ mono-test exhibited adequate analytical and clinical performance, showing good correlation with the Platelia™ *Aspergillus* Ag ELISA assay. The single-sample, semi-automated test is user-friendly, allowing small laboratories to perform the test on demand without the need for batch evaluations, providing a useful solution for timely diagnostic support for clinicians.

## 1. Introduction

*Aspergillus* spp. are ubiquitous filamentous fungi that cause invasive aspergillosis (IA), which remains a major cause of morbidity and mortality in immunocompromised patients. This is particularly true for high-risk individuals, such as those with hematological malignancies, solid organ transplants, or immune suppression due to immunotherapy [1]. Recently, IA has also been noted during viral respiratory infections, such as influenza and COVID-19, leading to new syndromic diagnoses referred to as IAPA (Influenza Associated Pulmonary Aspergillosis) and CAPA (COVID Associated Pulmonary Aspergillosis) [2,3,4]. CAPA has been reported in intensive care units (ICUs) with a median prevalence of 10.7%, showing significant variability between centers; moreover, CAPA is strongly associated with a significantly lower 90-day ICU survival rate [5].

The diagnosis of IA relies on a combination of clinical, radiological, and laboratory data [6]. Timely diagnosis is crucial to improving disease outcomes, especially given the high mortality associated with delays. Clinical and radiological signs of IA are often non-specific, necessitating additional diagnostic tests for confirmation. Traditional fungal diagnosis typically relies on culture tests, usually from respiratory samples, but circulating biomarkers are increasingly recommended in current guidelines [7]. Tests for galactomannan (GM) or β-D-glucan (BG) to detect circulating fungal antigens, as well as the detection of Aspergillus-specific DNA, have been integrated into clinical practice. However, these assays can present performance challenges [8]. Galactofuranose, the main antigenic component of GM, is produced by non-mammalian eukaryotic organisms and is relatively specific for Aspergillus species, although it is also present in Fusarium species [9]. This antigen is released during the tissue growth of invasive hyphae [10,11], providing insights into the invasive role of the observed mold. In contrast, BG is a pan-fungal antigen present in the cell walls of many yeasts and molds and is now considered in the diagnostic workflow for invasive candidiasis.

Several commercial tests for circulating galactomannan (GM) levels are available. The Platelia *Aspergillus* Galactomannan (PA) assay (Bio-Rad Laboratories, Richmond, CA, USA) is a sandwich ELISA that utilizes a highly efficient EB-A2 rat monoclonal antibody, capable of detecting 0.5 to 1 ng of the galactofuranose epitope. PA is the most widely recognized diagnostic test for GM antigen detection, with numerous published studies demonstrating its clinical and analytical performance. In addition, two novel Aspergillus lateral flow assays (LFAs) are available and approved for detecting Aspergillus antigens in both serum and bronchoalveolar lavage (BAL) samples: the IMMY Sona Aspergillus Lateral Flow Assay (IMMY, Norman, OK, USA) and the OLM Lateral Flow Device (OLM Diagnostics, Newcastle Upon Tyne, UK). While rapid and easy to use, LFA tests do not provide quantitative results.

In this study, we compared the performance of a new *Aspergillus* Galactomannan Ag Vir-CLIA^®^ mono-test (VA) with the established PA. This innovative test employs chemiluminescence detection and operates on an automated analyzer developed by Vircell. The system allows for single-sample runs, reducing the time to result and optimizing the diagnostic process. The first evaluation of the *Aspergillus* Galactomannan Ag Vir-CLIA^®^ mono-test was performed in a Spanish study [12], where the authors showed good agreement with the Platelia *Aspergillus* Galactomannan assay.

The aim of the present study was to corroborate and confirm the concordance between the two galactomannan antigen tests, with a particular focus on the evaluation of respiratory and serum samples. Unlike previous studies, which have primarily reported the overall results obtained from both types of samples analyzed, our study specifically compares the performance of the VA and PA assays in both biological matrices. We explored the degree of agreement between the two tests, considering the distinct characteristics of serum and respiratory samples. Furthermore, we analyzed the quantitative correlation between the assays, assessing reproducibility and consistency across the two sample types.

## 2. Materials and Methods

This study was conducted at the Microbiology Laboratory of the AOUI of Verona (Integrated University Hospital of Verona, Italy) on a group of 280 patients with hematological malignancy or patients with possible or probable invasive aspergillosis (IA), according to the European Organization of Research and Treatment of Cancer/Mycoses Study Group (EORTC/MSG) criteria [13]. Enrolled patients were recovered at the (Azienda Ospedaliera Universitaria Integrata) AOUI of Verona, a university hospital, between September 2019 and July 2020. During this period, clinical samples were collected and analyzed for the detection of GM at the Clinical Microbiology Laboratory.

Patients and clinical data were collected prospectively, and the GM detection was performed by the Platelia^TM^ *Aspergillus* Ag ELISA kit (Bio-Rad) according to the manufacturer’s instructions. Briefly, microwell plates coated with monoclonal anti-GM rat antibody were incubated with pre-treated clinical samples and conjugate reagent (peroxidase-linked monoclonal antibody). After a washing step, a 3,3′,5,5′—tetramethylbenzidine (TMB) chromogen was added, followed by further incubation. The reaction was stopped by adding an acid solution, and the absorbance was read at 450 nm. Serum samples with an index value ≥0.5 were considered positive for the GM antigen, while respiratory samples and biopsy were considered positive with an index value ≥1. Index values between 0.4 and 0.5 were considered doubtful, and an index value <0.4 was considered negative.

The residual sample’s volume was collected and analyzed by the *Aspergillus* Galactomannan Ag VirCLIA^®^ mono-test (Vircell) according to the manufacturer’s instructions. Briefly, after a manual pre-treatment step, similar to the Platelias’ pre-treatment step, serum was automatically transferred in a single strip that contains the negative control and calibrators together for each sample. Furthermore, Luminol, a chemiluminescent substrate, was used instead of TMB (3,3,5,5’-tetramethylbenzidine) to reveal the target quantitatively. The analysis was performed in an automated analyzer named Thunderbolt, developed by Vircell. The threshold for positivity was an index of 0.2 for both serum and respiratory samples, as proposed by the manufacturing company. Index values between 0.16 and 0.2 were considered doubtful, and index values <0.16 were considered negative.

The samples were analyzed in duplicate by both PA and VA on the same day or after being stored at −20 °C for up to a month.

To evaluate the reproducibility of the assay, two pools of 5 highly positive and 5 negative sera were, respectively, assembled. Each pool was tested for 12 consecutive runs.

### 2.1. Statistical Analysis

The patients’ and samples characteristics were reported by descriptive statistics. Continuous variables are expressed as medians, with interquartile range and categorical variables as number and percent values. All analyses, including the evaluation of VA accuracy, were performed using the software STATA for Windows version 16.0 (StataCorp LLC).

### 2.2. Ethical Statement

This study was approved by the institutional ethics committee (Comitato etico per la sperimentazione clinica Verona-Rovigo) 2479CESC—Studio Clinico: Evaluation of Vircell GALACTOMANNAN test in chemiluminescence method—Cod Prot: SP19002 (CE prot. 3457 del 20 January 2020).

## 3. Results

To compare the performance of VA, 282 samples were prospectively collected from daily PA applications between September 2019 and July 2020.

**Figure 1:**
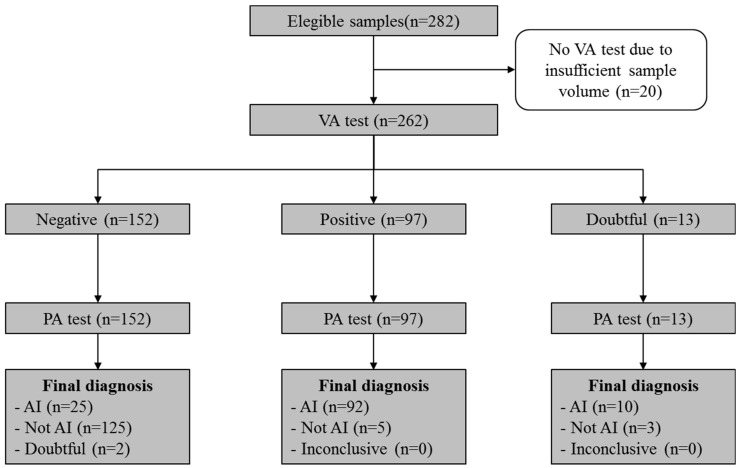
Study flow diagram reported as STARD guidelines [14].

Hematological patients and individuals with suspected invasive aspergillosis were included in this study. A total of 280 patients were enrolled for GM monitoring following ESCMID guidelines (sex ratio M/F, 180/100 (1.8); median (interquartile range) age, 63 (53–73)). Evaluation with the VA was not possible for 20 samples due to insufficient volume (Figure 1).

Within 262 VA-tested samples, 187 were sera, 73 were bronchoalveolar washes, 1 was biopsy, and 1 pleural fluid (Table 1). Patients’ underlying conditions are reported. As Figure 1 shows, 15 (5,7%) samples reported doubtful results: 2 and 13 samples with the PA (0.41–0.42 index) and with the VA (0.160–0.198 index), respectively. The first evaluation (Analysis A) was performed on 247 samples, excluding the 15 samples with doubtful results. Analysis B was then performed on all 262 samples, including doubtful samples as negative results (both at VA and PA tests). Finally, Analysis C was performed on all 262 samples but it included doubtful samples as positive results (both at VA and PA tests).

The results from Analysis A demonstrated that 217 out of 247 determinations (87.85%) were concordant between both techniques, comprising 92 positive and 125 negative results (Table 2). In Analysis B, 222 determinations (84.7%) were concordant, with 92 positive and 130 negative results, while Analysis C showed 227 concordant determinations (86.6%), consisting of 102 positive and 125 negative results (Table 3 and Table 4).

**Figure 2:**
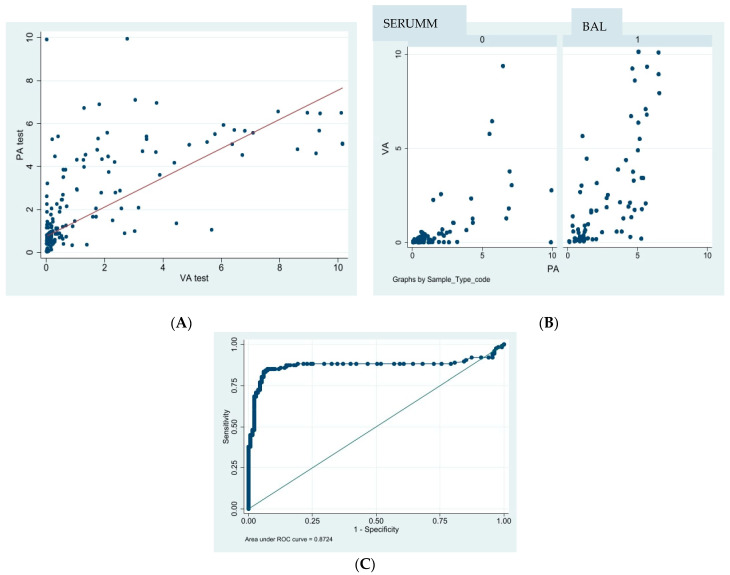
Quantitative correlation of Galactomannan Antigen index with PA and VA. (**A**) All the sample results are considered. (**B**) Result from serum and BAL (Bronchoalveolar lavage) are separately considered. (**C**) Analisis A—ROC curve.

The main accuracy characteristics of the VA are summarized in Table 5. The ROC curves are showed in Figure 2. The degree of agreement between the two methods was assessed using Cohen’s kappa coefficient (κ). The values reported in Table 6 indicate a “substantial” agreement between the tests.

As shown in Figure 2, quantitative correlation was evaluated in two conditions: all types of samples (A) and discrimination between serum samples and BAL (B). No quantitative correlation factor was found.

**Figure 3:**
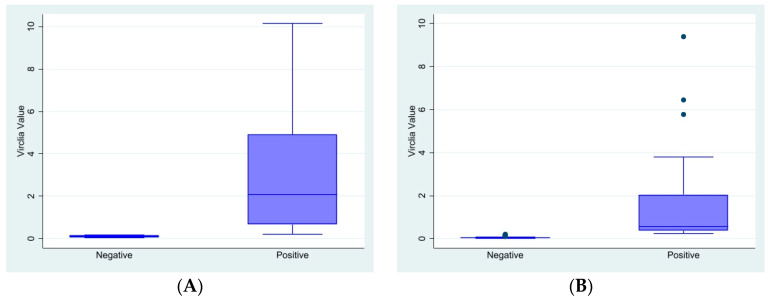
Distribution of VA data according PA interpretation, SERUM and BAL separately considered. (**A**) BAL. Positive: PA index value ≥ 1; negative: PA index value < 1. (**B**) Serum. Positive: PA index value ≥ 0.5; negative: PA index value < 0.4.

The distribution of VA quantitative results considering PA categorical results is reported in Figure 3.

An evaluation of the intra-assay imprecision of the *Aspergillus* Galactomannan antigen VirCLIA, conducted to validate the method’s repeatability, yielded a coefficient of variation (CV) of 0.13 for a low-value serum pool (12 repeats; mean value, 0.017 ± 0.003) and 0.18 for a very high-value serum pool (12 repeats; mean value, 2.94 ± 0.401), respectively.

## 4. Discussion

Our study demonstrated a strong agreement between the *Aspergillus* Galactomannan Ag VirCLIA^®^ mono-test (Vircell S.L.) and the Platelia™ *Aspergillus* Ag ELISA assay (Bio-Rad), with an overall concordance of 87.8%, and sensitivities and specificities of 78.6% and 96.2%, respectively (Table 5 and Table 6). These findings align with those reported by Calero et al., who observed 89% agreement when considering doubtful samples as positive and 93.7% when considered negative [13]. Results near the cutoff value can be challenging to interpret, often indicating the onset of infection or potential false positives in ELISA procedures. Our analyses of VA results included various approaches, showing the highest concordance when excluding doubtful results or treating them as positive (Table 5 and Table 6). However, unlike the study by Calero et al., in which the data were not stratified by sample type, our study distinctly analyzed the performance of the two assays in serum and BAL (bronchoalveolar lavage) samples. Notably, we observed significant differences in the performance of the tests when comparing these two types of biological materials. Our results indicate better agreement and more reliable outcomes with respiratory samples (BAL) compared to serum, suggesting that BAL may be a more suitable matrix for GM detection in the context of invasive aspergillosis (IA) in our setting. This differs from Calero’s study, which used both serum and BAL samples, but did not provide a specific direct comparison between these two sample types. The manufacturer’s guidelines recommend retesting doubtful results to rule out technical errors; however, our study’s design limited the availability of serum for repeat testing, which may have affected the interpretation of these results.

Quantitative assessments of PA have been widely studied and are known to serve as effective indicators of treatment response in hematological IA [15]. Despite analyzing quantitative results from both tests, our study found no significant mathematical correlation between the VA and PA index data for either serum or BAL samples (Figure 2). This lack of correlation may be partly due to the limited sample size, which is a key limitation of our study. A larger sample size would likely provide more definitive insights into this clinical question. Although assessing the quantitative impact of VA was not the primary focus of our study, it remains an important area for future research, as quantitative data are crucial for clinicians to evaluate patient progression.

Both serum and BAL samples were used to evaluate the performance of the new GM test. Indeed, BAL samples are particularly useful in non-hematological patients, where serum GM levels may be undetectable despite a high probability of AI [16]. While GM detection on serum is particularly effective in neutropenic leukemia patients, its sensitivity may decrease in high-risk hematological patients undergoing antifungal prophylaxis or in other immunocompromised non-neutropenic individuals. GM indices in BAL have instead been identified as effective biomarkers for the diagnosis of AI, and specific cutoffs have been proposed for the PA to differentiate between infection and colonization [17]. Specific cutoffs for the VA are currently not available, but their definition could help clinicians to assess pathogen invasiveness more effectively.

One limitation of our study is the absence of culture results or Aspergillus DNA findings from the same samples used for GM detection. However, the concurrent testing of both PA and VA tests minimized potential bias related to sample storage and processing.

The diagnosis of invasive fungal infections remains a critical challenge, often delayed due to limitations in current methodologies. Traditional culture-based procedures, supplemented by microscopic examinations, still dominate laboratory diagnostics for IA. GM detection in serum and respiratory samples is now widely accepted as a primary biomarker, allowing for timely intervention and reduced inappropriate therapies. While commercial Aspergillus nucleic acid amplification tests (NAATs) are available, many lack standardization [18].

Despite some limitations in performance across different at-risk populations, GM testing remains the most prevalent diagnostic method. The Platelia™ *Aspergillus* Ag ELISA assay, FDA-approved since 2003, is the most widely used, detecting GM produced during Aspergillus invasive growth with a rat monoclonal IgM antibody (EB-A2). Another test, the Aspergillus-specific galactomannoprotein ELISA (GP ELISA; Euroimmun, Lübeck, Germany), employs a different monoclonal antibody (IgG3 JF5), which appears to minimize cross-reactivity with other yeasts and molds like *Cryptococcus*, *Penicillium marneffei* [19], or *Fusarium* [9,20]. Both tests rely on ELISA techniques, necessitating sample batching, which can extend turnaround times (TAT) for medical reporting and treatment [21]. In contrast, lateral flow assays (LFAs) offer rapid results but are limited in automation and are best suited for low-volume labs. While LFAs could serve as alternatives for GM detection in such settings, comprehensive data supporting their efficacy remain sparse.

In conclusion, this work confirms that PA has long been the reference method, but its reliance on ELISA technology complicates the analysis of single or few samples. The VirClia^®^ (VA) system, however, meets clinical laboratory needs by facilitating analysis within hours, even with limited samples, demonstrating that performance even on biological respiratory samples is very good. Additionally, the VA system requires minimal sample handling, making it an advantageous option for IA diagnosis in clinical microbiology laboratories.

## Figures and Tables

**Table 1:** Main characteristics of study patients and samples.

	N (%)
**Patients**	**280**
Males	180 (64)
Females	100 (36)
**Patients underlying conditions**	
Hematological disease and Bone marrow transplants	135 (48)
ICU and surgical patients	75 (26.5)
Infectious disease and other medical disease	54 (19)
Other clinical conditions	18 (6)
**Collected Samples**	**282**
Samples Virclia skipped	20 (7)
**Effective Compared Samples**	**262 (93)**
Serum	187 (71.3)
Bronchoalveolar washes	73 (27.9)
Biopsy	1 (0.4)
Pleuric fluid	1 (0.4)

**Table 2:** Distribution of results in Analysis A.

		Platelia (PA)		Total
		Positive	Negative	
Virclia (VA)	positive	92	5	97
	negative	25	125	150
Total		117	130	247

**Table 3:** Distribution of results in Analysis B.

		Platelia (PA)		Total
		Positive	Negative	
Virclia (VA)	positive	92	5	97
	negative	35	130	165
Total		127	135	262

**Table 4:** Distribution of results in Analysis C.

		Platelia (PA)		Total
		Positive	Negative	
Virclia (VA)	positive	102	8	110
	negative	27	125	152
Total		129	133	262

**Table 5:** Accuracy characteristics of VA obtained from Analysis A, B, and C, evaluating all, BAL/BAS and serum samples. The prevalence was 47%, 48%, and 49% for Analysis A, B and C, respectively, based on Platelia results. Legend: BAL/BAS: bronchoalveolar lavage/bronchial aspirates; PPV: positive predictive value; NPV: negative predictive value.

	Analysis A		
	All Samples	BAL/BAS	SERUM
**Sensitivity**	78.6% [70.1–85.7%]	91.8% [81.9–97.3%]	63% [48.7–75.7%]
**Specificity**	96.2% [91.3–98.7%]	57.1% [18.4–90.1%]	98.4% [94.2–99.8%]
**PPV**	94.8% [88.4–98.3%]	94.9% [85.9–98.9%]	94.4% [81.3–99.3%]
**NPV**	83.3% [76.4–88.9%]	44.4% [13.7–78.8%]	85.8% [78.9–91.1%]
**ROC Area**	0.874 [0.833–0.915]	0.74 [0.54–0.94]	0.80 [0.74–0.87]
	**Analysis B**		
	**All samples**	**BAL/BAS**	**SERUM**
**Sensitivity**	72.4% [63.8–80.0%]	84.8% [73.9–92.5%]	57.6% [44.1–70.4%]
**Specificity**	96.3% [91.6–98.8%]	57.1% [18.4–90.1%]	98.4% [94.5–99.8%]
**PPV**	94.8% [88.4–98.3%]	94.9% [85.9–98.9%]	94.4% [81.3–99.3%]
**NPV**	78.8% [71.8–84.8%]	28.6% [8.39–58.1%]	83.4% [76.5–89%]
**ROC Area**	0.844 [0.802–0.886]	0.71 [0.507–0.913]	0.78 [0.716–0.845]
	**Analysis C**		
	**All samples**	**BAL/BAS**	**SERUM**
**Sensitivity**	79.1% [71.0–85.7%]	92.4% [83.2–97.5%]	63.9% [50.6–75.8%]
**Specificity**	94.0% [88.5–97.4%]	57.1% [18.4–90.1%]	96% [91–98.7%%]
**PPV**	92.7% [86.2–96.8%]	95.3% [86.9–99%]	88.6% [75.4–96.2%%]
**NPV**	82.2% [75.2–88.0%]	44.4% [13.7–78.8%]	84.6% [77.6–90.1%]
**ROC Area**	0.865 [0.825–0.906]	0.74 [0.54–0.94]	0.8 [0.737–0.863]

**Table 6:** Agreement data from Analysis A, B, C.

	Analysis A	Analysis B	Analysis C
Agreement (%)	87.85	84.73	86.64
Cohen’s kappa coefficient (r)	0.75	0.69	0.73
Standard error	0.06	0.06	0.06
95% CI	0.63–0.88	0.57–0.81	0.61–0.85

## Data Availability

The data presented in this study are available on request from the corresponding author due to privacy ethical statements.

## References

[1] Brown G.D., Denning D.W., Gow N.A.R., Levitz S.M., Netea M.G., White T.C. (2012). Hidden Killers: Human Fungal Infections. Sci. Transl. Med..

[2] Verweij P.E., Rijnders B.J.A., Brüggemann R.J.M., Azoulay E., Bassetti M., Blot S., Calandra T., Clancy C.J., Cornely O.A., Chiller T. (2020). Review of influenza-associated pulmonary aspergillosis in ICU patients and proposal for a case definition: An expert opinion. Intensive Care. Med..

[3] Koehler P., Cornely O.A., Böttiger B.W., Dusse F., Eichenauer D.A., Fuchs F., Hallek M., Jung N., Klein F., Persigehl T. (2020). COVID-19 associated pulmonary aspergillosis. Mycoses.

[4] Dimopoulos G., Almyroudi M.-P., Myrianthefs P., Rello J. (2021). COVID-19-Associated Pulmonary Aspergillosis (CAPA). J. Intensiv. Med..

[5] Prattes J., Wauters J., Giacobbe D.R., Salmanton-García J., Maertens J., Bourgeois M., Reynders M., Rutsaert L., Van Regenmortel N., Lormans P. (2022). Risk factors and outcome of pulmonary aspergillosis in critically ill coronavirus disease 2019 patients—A multinational observational study by the European Confederation of Medical Mycology. Clin. Microbiol. Infect..

[6] Patterson T.F., Thompson G.R., Denning D.W., Fishman J.A., Hadley S., Herbrecht R., Kontoyiannis D.P., Marr K.A., Morrison V.A., Nguyen M.H. (2016). Practice Guidelines for the Diagnosis and Management of Aspergillosis: 2016 Update by the Infectious Diseases Society of America. Clin. Infect. Dis..

[7] Ullmann A.J., Aguado J.M., Arikan-Akdagli S., Denning D.W., Groll A.H., Lagrou K., Lass-Flörl C., Lewis R.E., Munoz P., Verweij P.E. (2018). Diagnosis and management of Aspergillus diseases: Executive summary of the 2017 ESCMID-ECMM-ERS guideline. Clin. Microbiol. Infect..

[8] Patterson T.F., Donnelly J.P. (2019). New Concepts in Diagnostics for Invasive Mycoses: Non-Culture-Based Methodologies. J. Fungi..

[9] Tortorano A.M., Esposto M.C., Prigitano A., Grancini A., Ossi C., Cavanna C., Cascio G.L. (2012). Cross-Reactivity of Fusarium spp. in the *Aspergillus* Galactomannan Enzyme-Linked Immunosorbent Assay. J. Clin. Microbiol..

[10] Latgé J.-P., Chamilos G. (2019). Aspergillus fumigatus and Aspergillosis in 2019. Clin. Microbiol. Rev..

[11] Fontaine T., Latgé J.-P. (2020). Galactomannan Produced by *Aspergillus. fumigatus*: An Update on the Structure, Biosynthesis and Biological Functions of an Emblematic Fungal Biomarker. J. Fungi..

[12] Calero A.L., Alonso R., Gadea I., Vega M.D.M., García M.M., Muñoz P., Machado M., Bouza E., García-Rodríguez J. (2022). Comparison of the Performance of Two Galactomannan Detection Tests: Platelia *Aspergillus* Ag and *Aspergillus* Galactomannan Ag Virclia Monotest. Microbiol. Spectr..

[13] Donnelly J.P., Chen S.C., Kauffman C.A., Steinbach W.J., Baddley J.W., Verweij P.E., Clancy C.J., Wingard J.R., Lockhart S.R., Groll A.H. (2020). Revision and Update of the Consensus Definitions of Invasive Fungal Disease From the European Organization for Research and Treatment of Cancer and the Mycoses Study Group Education and Research Consortium. Clin. Infect. Dis..

[14] Bossuyt P.M., Reitsma J.B., Bruns D.E., Gatsonis C.A., Glasziou P.P., Irwig L., Lijmer J.G., Moher D., Rennie D., de Vet H.C.W. (2015). STARD 2015: An Updated List of Essential Items for Reporting Diagnostic Accuracy Studies. Radiology.

[15] Lanternier F., Seidel D., Pagano L., Styczynski J., Mikulska M., Pulcini C., Maertens J., Munoz P., Garcia-Vidal C., Rijnders B. (2020). Invasive pulmonary aspergillosis treatment duration in haematology patients in Europe: An EFISG, IDWP-EBMT, EORTC-IDG and SEIFEM survey. Mycoses.

[16] Zhou W., Li H., Zhang Y., Huang M., He Q., Li P., Zhang F., Shi Y., Su X. (2017). Diagnostic Value of Galactomannan Antigen Test in Serum and Bronchoalveolar Lavage Fluid Samples from Patients with Nonneutropenic Invasive Pulmonary Aspergillosis. J. Clin. Microbiol..

[17] Kono Y., Tsushima K., Yamaguchi K., Kurita N., Soeda S., Fujiwara A., Sugiyama S., Togashi Y., Kasagi S., To M. (2013). The utility of galactomannan antigen in the bronchial washing and serum for diagnosing pulmonary aspergillosis. Respir. Med..

[18] Zhang S.X., Babady N.E., Hanson K.E., Harrington A.T., Larkin P.M.K., Leal S.M., Luethy P.M., Martin I.W., Pancholi P., Procop G.W. (2021). Recognition of Diagnostic Gaps for Laboratory Diagnosis of Fungal Diseases: Expert Opinion from the Fungal Diagnostics Laboratories Consortium (FDLC). J. Clin. Microbiol..

[19] Huang Y.-T., Hung C.-C., Liao C.-H., Sun H.-Y., Chang S.-C., Chen Y.-C. (2007). Detection of Circulating Galactomannan in Serum Samples for Diagnosis of *Penicillium marneffei* Infection and Cryptococcosis among Patients Infected with Human Immunodeficiency Virus. J. Clin. Microbiol..

[20] Thornton C.R. (2008). Development of an Immunochromatographic Lateral-Flow Device for Rapid Serodiagnosis of Invasive Aspergillosis. Clin. Vaccine. Immunol..

[21] Dichtl K., Seybold U., Ormanns S., Horns H., Wagener J. (2019). Evaluation of a Novel *Aspergillus.* Antigen Enzyme-Linked Immunosorbent Assay. J. Clin. Microbiol..

